# The oral manifestations and related mechanisms of COVID-19 caused by SARS-CoV-2 infection

**DOI:** 10.3389/fncel.2022.1006977

**Published:** 2023-01-04

**Authors:** Weiming Lin, Feng Gao, Xia Wang, Nianhong Qin, Xianxiong Chen, Kin Yip Tam, Chengfei Zhang, Mingxia Zhang, Ou Sha

**Affiliations:** ^1^Shenzhen University Medical School, Shenzhen, China; ^2^School of Dentistry, Shenzhen University Medical School, Shenzhen, China; ^3^Faculty of Health Sciences, University of Macau, Macau, Macau SAR, China; ^4^School of Dentistry, The University of Hong Kong, Hong Kong, Hong Kong SAR, China; ^5^The Third People’s Hospital of Shenzhen, Shenzhen, China

**Keywords:** COVID-19, SARS-CoV-2, oral manifestations, ACE2 receptor, TMPRSS2, influential factors

## Abstract

Coronavirus disease 2019 (COVID-19) was reported to be associated with severe acute respiratory syndrome coronavirus-2 (SARS-CoV-2) infection, and patients present mostly with respiratory symptoms. There have been an increasing number of reports on oral manifestations, and some of these signs are informative in terms of identifying SARS-CoV-2 infection. The goal of present study was to review and synthesize the clinical characteristics and underlying mechanisms of COVID-19 oral manifestations, as well as to evaluate the factors influencing SARS-CoV-2 infectivity, in order to conduct further in-depth investigations and help clinicians diagnose COVID-19 patients exhibiting oral symptoms.

## 1. Introduction

More than 110.38 million people have been affected by the coronavirus disease 2019 (COVID-19) outbreak, which has now spread to 224 countries and killed more than 2.44 million people (Chan et al., 2020; Harrison et al., 2020). The International Committee on Classification of Viruses has named the virus formally severe acute respiratory syndrome coronavirus-2 (SARS-CoV-2) (Gorbalenya et al., 2020). The genome of SARS-CoV-2 isa linear single-stranded sense RNA, containing 14 open reading frames (ORFs), which encode proteins including spike protein (S), envelope protein (E), membrane protein (M), and nucleocapsid protein (N) (Kim et al., 2020; Papageorgiou and Mohsin, 2020; Arya et al., 2021; Yan et al., 2022). S protein is responsible for the viral infectivity and affinity for host cells. It is necessary for receptor binding and encouraging the fusion of the virus and cell membranes during viral invasion of host cells (Jackson et al., 2022).

The most common signs of COVID-19 are fever, coughing, dyspnea, and in severe cases, even death. More cases of COVID-19 extrapulmonary symptoms, including oral signs, are being reported (Chen N. et al., 2020; Rodriguez-Morales et al., 2020; Wang D. et al., 2020; da Rosa Mesquita et al., 2021). According to statistics, two-thirds of COVD-19 patients have at least one oral symptom (El Kady et al., 2021), and roughly one-third of patients have dysgeusia as their initial symptom (Biadsee et al., 2020). Dysgeusia and xerostomia are the most common oral manifestations of COVID-19 patients. The former one refers to patients’ inability to identify the taste of food or drink, and the latter one means that patients cannot smell the odor of food or drink. Additionally, the majority of patients with oral symptoms exhibited anomalies in their oral cavities 3 months after being released from the hospital, indicating that oral symptoms might be one of COVID-19’s aftereffects (Gherlone et al., 2021). The oral symptoms of COVID-19 patients have been the subject of numerous studies, and the appearance of oral symptoms is generally viewed as a reminder of viral infection.

The purpose of the current study was to review the most recent research on SARS-CoV-2 infection in the oral cavity.

## 2. Methods

The PubMed, Scopus and Web of Science databases were used for literature search to determine the literature related to the oral manifestations and related mechanisms of COVID-19. The keywords used were: “oral manifestations,” “dysgeusia,” “xerostomia,” “oral mucosal lesions,” “central nervous system,” “peripheral nervous system,” “Olfactory dysfunction,” “entry factors,” “ACE2,” “TMPRSS2,” “Furin,” “cathepsin,” “mechanisms,” “influential factors,” “SARS-CoV-2,” “Corona virus disease pandemic,” “COVID-19,” “2019-nCoV.” Studies were limited to those in English language included in PubMed, Scopus and Web of Science databases. Exclusion criteria included non-English language studies and those not included in PubMed, Scopus and Web of Science databases. According to the exclusion and inclusion criteria, all studies were independently screened by two reviewers, first by the title/abstract, and then the full text. Data or the research results extracted from the included studies were used for analysis.

## 3. Oral manifestations of COVID-19

**FIGURE 1:**
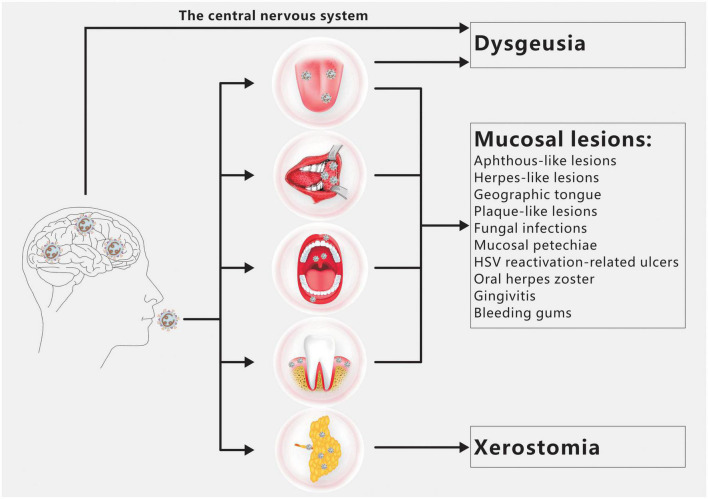
Overview of COVID-19 oral manifestations. Dysgeusia, oral mucosal lesions and xerostomia are the main oral symptoms of COVID-19. SARS-CoV-2 infection of the CNS and/or taste buds is the main cause of dysgeusia. Oral mucosal lesions include aphthous-like lesions, herpes-like lesions, geographic tongue, plaque-like lesions, fungal infections, mucosal petechiae, HSV reactivation-related ulcers, oral herpes zoster, gingivitis and bleeding gums. The primary cause of xerostomia is SARS CoV-2 infection of salivary gland acinar cells and ductal epithelium.

Dysgeusia, xerostomia, and oral mucosal lesions are the three oral symptoms of COVID-19 most frequently observed (Figure 1; Amorim dos Santos et al., 2021a). There are a number of additional oral symptoms, such as facial paralysis, trigeminal neuralgia, Melkersson-Rosenthal syndrome, macroglossia, anomalies of the temporomandibular joint, pain and swelling of the masticatory muscles, etc., although these secondary symptoms have not been widely documented (Amorim dos Santos et al., 2021b; El Kady et al., 2021; Farid et al., 2022; Sharma et al., 2022).

### 3.1. Dysgeusia

Different sources have reported on the prevalence of dysgeusia, and anosmia frequently coexists with it (Sharma et al., 2022). However, Patients occasionally struggled to discern between dysgeusia and anosmia (El Kady et al., 2021). One of the earliest signs of SARS-CoV-2 infection has been reported as dysgeusia, which was typically seen in female patients with mild to moderate COVID-19 (Amorim dos Santos et al., 2021a; Iranmanesh et al., 2021). However, dysgeusia in COVID-19 patients is not substantially correlated with patients’ age, gender, or employment (El Kady et al., 2021). Dysgeusia can manifest in the majority of patients within 5 days of receiving a COVID-19 diagnosis, and it typically lasts for 2 weeks, or up to 4 weeks in more severe cases (Amorim dos Santos et al., 2021a). It was discovered that the degree of dysgeusia was strongly correlated with the severity of COVID-19, and that severe dysgeusia served as a warning sign (Amorim dos Santos et al., 2021a; Kumar, 2021). It is interesting that among COVID-19 patients, there was no statistically significant difference in the alterations of the tastes of sour, sweet, salty, or spicy (Biadsee et al., 2020).

### 3.2. Xerostomia

Saliva secretion is frequently impaired after SARS-CoV-2 infection, and xerostomia is the most common oral symptom in COVID-19 patients (Amorim dos Santos et al., 2021b). Initially, Chen L. et al. (2020) found that 46.3% of patients had xerostomia, with no discernible gender difference but severe cases appeared to be more prone to develop. Patients with xerostomia frequently experienced various symptoms along with their main complaint of dry mouth, such as a burning feeling, dysgeusia, angular stomatitis, and dysphagia (Biadsee et al., 2020; Eghbali Zarch and Hosseinzadeh, 2021; Tsuchiya, 2021). Despite not lethal, xerostomia can have a substantial impact on a patient’s life quality and dental health (Tsuchiya, 2021). Notably, sialadenitis may also be found in patients. In a case described by Fisher et al. (2021) the patient experienced symptoms of both acute bacterial suppurative parotitis and viral parotitis. Lechien et al. (2020) reported 3 cases of COVID-19-related parotitis. All three patients sought care for unilateral ear pain and retromandibular edema, and magnetic resonance imaging (MRI) indicated the occurrence of intracarotid lymphadenitis. Capaccio et al. (2020) demonstrated that the COVID-19-related acute parotitis may be one of the virus’s first symptoms. Amorim dos Santos et al. (2021b) summarized numerous reports of sialadenitis, and discovered that unilateral parotid gland lesions were frequently recorded. Furthermore, the most frequent oral sequelae are xerostomia and dilation of salivary ducts, indicating that there was a significant inflammatory response in the salivary glands of COVID-19 patients (Gherlone et al., 2021).

### 3.3. Oral mucosal lesions

Less frequently occurring than dysgeusia and xerostomia, oral mucosal lesions were seen in about 20.5% of COVID-19 patients (Amorim dos Santos et al., 2021b). The majority of patients reportedly experienced oral mucosal lesions within 10 days of infection, and they were often treated within 1–3 weeks after receiving photobiomodulation therapy (PBMT) and/or antiviral medication (Amorim dos Santos et al., 2021a; Brandão et al., 2021). Elderly, long-term hospitalized, unhygienic, or diabetic people are more likely to have oral mucosal lesions, and these individuals also tend to have more severe, long-lasting, and wide-ranging oral lesions (Iranmanesh et al., 2021). In addition, it had been stated that aphthous-like lesions, herpes-like lesions, Kawasaki-like disease (geographic tongue), plaque-like lesions, fungal infections (candidiasis and mucormycosis), mucosal petechiae, herpes simplex virus (HSV) reactivation-related ulcers, oral herpes zoster, gingivitis, and bleeding gums are frequently seen (Amorim dos Santos et al., 2021a; Iranmanesh et al., 2021; Orilisi et al., 2021; Sharma et al., 2022). The most frequent lesions, according to a recent study, are aphthous-like lesions, which are distinguished by many, round or irregular shapes, an erythematous halo, a surface coated in a purulent membrane, a white pseudomembrane, etc (Brandão et al., 2021). It is noteworthy that patients with oral lesions resembling Kawasaki are more likely to develop severe COVID-19 or require hospital admission (Erbaş et al., 2022).

## 4. The mechanisms of oral manifestations

### 4.1. The expression of entry factors and the entry pathways of SARS-CoV-2 in oral cavity

Severe acute respiratory syndrome coronavirus-2 assaults host cells through interacting with angiotensin-converting enzyme 2 (ACE2) receptors, inducing inflammatory responses in corresponding tissues and organs, which is similar to SARS-CoV. Additionally, the S protein containing S1 and S2 domains can be cleaved by Furin or transmembrane serine protease 2 (TMPRSS2) to accelerate the virus-cell membrane fusion and increase the viral tropism to organs, which may justify why SARS-CoV-2 has a higher infection rate than SARS-CoV. There are also some airway proteases such as TMPRSS4, TMPRSS11A, TMPRSS11E, TMPRSS13, human airway trypsin-like protease (HAT), matriptase, differentially expressed in squamous cell carcinoma 1 (DESC1), secreted neutrophil elastase, etc. appear to contribute to respiratory virus infection (Laporte and Naesens, 2017; Zou et al., 2020; Jackson et al., 2022). Therefore it is necessary to summarize the expression of SARS-CoV-2 entry factors in distinct structures of oral cavity in order to predict the infection of oral cavity by the virus and to reveal the mechanism of oral symptoms in COVID-19 patients (Table 1).

**TABLE 1:** The expression and function of SARS-CoV-2 entry factors in varying structures of oral cavity.

Entry factor	Function	Location	Sample source	Expression of the factor	References
ACE2	SARS-CoV-2 receptor	Tongue (taste bud)	Human	In the type II and III taste cells of the fungiform and circumvallate papillae.	Sakaguchi et al., 2020; Okada et al., 2021; Drozdzik and Drozdzik, 2022
			SD rat	In the taste bud cells of the fungiform papillae.	Park et al., 2022
		Tongue (mucosa)	Human	In the keratinized stratified squamous epithelium and endothelial cells. Oral tongue >buccal and gingival tissues.	Sakaguchi et al., 2020; Xu H. et al., 2020; Huang et al., 2021; Okada et al., 2021; Sawa et al., 2021; Drozdzik and Drozdzik, 2022
			SD rat	In the mature keratinocytes in the suprabasal layer of the squamous epithelium. Ventral mucosa >dorsal of the tongue, sporadically in the lamina propria and muscle.	Park et al., 2022
		Salivary glands	Human	Similar in men and women. Salivary glands >oral cavity mucosa. Minor salivary glands >parotid >submandibular >sublingual glands. Salivary gland: Ducts, serous, and mucous acini clusters. Parotid gland: Serous cells, ductal epithelium, and adipocytes. Sublingual gland and buccal gland: Serous demilunes, ductal epithelium, and endothelial cells. Submandibular glands: Serous cells, ductal epithelium, and saliva of the ductal cavity.	Baughn et al., 2020; Sakaguchi et al., 2020; Huang et al., 2021; Matuck et al., 2021; Okada et al., 2021; Drozdzik and Drozdzik, 2022
			SD rat	Strongly expressed in all salivary gland ductal cells. Strongly expressed in the acinar cell of parotid gland, Sporadically present in the submandibular and minor salivary gland.	Park et al., 2022
		Lips	Human	In the non-keratinized stratified squamous epithelia in labial mucosa. In the mucous acini and serous acini in labial gland.	Sawa et al., 2021; Drozdzik and Drozdzik, 2022
		Gingiva	Human	In the sulcular epithelium and periodontal pocket epithelium. In the spinous–basal cell layer, but not the epithelial surface and horny layer. Suprabasal >basal cells.	Sakaguchi et al., 2020; Huang et al., 2021; Drozdzik and Drozdzik, 2022
		Buccal mucosa	Human	In non-keratinized stratified squamous epithelia. Suprabasal >basal cells.	Huang et al., 2021; Okada et al., 2021; Sawa et al., 2021; Drozdzik and Drozdzik, 2022
			SD rat	Intermediate layer >basal and superficial layers. Not expressed in immature keratinocytes or in the basal layers of the squamous epithelia.	Park et al., 2022
		Soft palate	Human	Suprabasal >basal cells.	Huang et al., 2021; Drozdzik and Drozdzik, 2022
			SD rat	Weak expression in a few superficial keratinocytes. Not expressed in immature keratinocytes/basal layers of the epithelia/the taste buds.	Park et al., 2022
		Tonsil	Human	In the tonsillar crypt. Suprabasal >basal cells.	Huang et al., 2021
		Blood vessel	Human	In arterial/venous endothelial cells and arterial smooth muscle cells in oral cavity.	Hamming et al., 2004; Okada et al., 2021
			SD rat	Not in venules/arterioles. Strongly in the capillaries of the salivary glands.	Park et al., 2022
TMPRSS2	Cleave S2’ site of S protein	Tongue (taste bud)	Human	Strongly in the taste bud cells of the fungiform papilla.	Sakaguchi et al., 2020; Okada et al., 2021; Drozdzik and Drozdzik, 2022
			SD rat	In the taste bud cells of the fungiform papillae. Strongly in the pore cells of the fungiform papillae.	Park et al., 2022
		Tongue (mucosa)	Human	In the keratinized stratified squamous epithelia and endothelial cells as well as tongue coating of human (mainly in the stratum granulosum and stratum spinosum). Suprabasal >basal cells.	Sakaguchi et al., 2020; Huang et al., 2021; Okada et al., 2021; Sawa et al., 2021; Drozdzik and Drozdzik, 2022
			SD rat	Ventral mucosa >dorsal mucosa. Strongly in the intermediate layer of the squamous epithelia. Rarely in basal cells. Strongly in the muscle layer mast cells.	Park et al., 2022
		Salivary glands	Human	Similar in men and women. Salivary glands >oral mucosa. Minor salivary glands >parotid >submandibular >sublingual glands. Salivary gland: Ducts epithelium. Serous acini >mucous acini Parotid gland: Serous cells, ductal epithelium, and adipocytes. Sublingual gland and buccal gland: Serous demilunes, ductal epithelium, and endothelial cells. Submandibular glands: Serous cells, ductal epithelium, and saliva of the ductal cavity.	Baughn et al., 2020; Sakaguchi et al., 2020; Huang et al., 2021; Matuck et al., 2021; Okada et al., 2021; Drozdzik and Drozdzik, 2022
			SD rat	Strongly expressed in all salivary gland ductal cells. Strongly expressed in the acinar cell of parotid gland, Sporadically present in the submandibular and minor salivary gland.	Park et al., 2022
		Lips	Human	In the non-keratinized stratified squamous epithelia in labial mucosa. In the mucous acini and serous acini in labial gland. Serous acini >mucous acini	Sawa et al., 2021; Drozdzik and Drozdzik, 2022
		Gingiva	Human	In the sulcular epithelium and periodontal pocket epithelium. In the spinous cell layer of epithelia, not basal layer.	Sakaguchi et al., 2020; Huang et al., 2021; Drozdzik and Drozdzik, 2022; Ohnishi et al., 2022
		Buccal mucosa	Human	In the non-keratinized stratified squamous epithelia. Suprabasal >basal cells.	Huang et al., 2021; Okada et al., 2021; Sawa et al., 2021; Drozdzik and Drozdzik, 2022
			SD rat	Intermediate layer >basal and superficial layers. Not expressed in immature keratinocytes or in the basal layers of the squamous epithelia.	Park et al., 2022
		Soft palate	Human	Increased suprabasal expression was observed when compared with the basal compartment.	Huang et al., 2021; Drozdzik and Drozdzik, 2022
			SD rat	Weak expression in a few superficial keratinocytes. Not expressed in immature keratinocytes or in the basal layers of the squamous epithelia. Not expressed in the taste buds of the soft palate mucosa.	Park et al., 2022
		Tonsil	Human	Suprabasal >basal	Huang et al., 2021
		Blood vessel	Human	Vascular endothelial cells.	Okada et al., 2021
			SD rat	Not in venules/arterioles. Strongly in the capillaries of the salivary glands.	Park et al., 2022
Furin	Cleave a multibasic site (Arg-Arg-Ala-Arg) located at the S1–S2 junction of S protein during biosynthesis and maturation of SARS-CoV-2 in the infected cell.	Tongue (taste bud)	Human	In the lower layers of the taste buds of the fungiform papillae.	Sakaguchi et al., 2020; Okada et al., 2021
		Tongue (mucosa)	Human	In the spinous and basal cell layers of the epithelium in a dotted pattern.	Sakaguchi et al., 2020; Okada et al., 2021
		Salivary glands	Human	Submandibular glands: In the serous cells and saliva of the ductal cavity, not in the ductal epithelium. Parotid gland: In the serous cells and ductal epithelium. Sublingual gland and the buccal gland: in the serous demilunes and ductal epithelium.	Sakaguchi et al., 2020; Okada et al., 2021
		Lips	Human	Furin was expressed in lips.	
		Gingiva	Human	In the spinous and basal cell layers.	Sakaguchi et al., 2020
		Buccal mucosa	Human	In the spinous and basal cell layers of the epithelium in a dotted pattern.	Okada et al., 2021
		Soft palate	Human	Furin was expressed in soft palate.	
		Tonsil	NA	NA	
		Blood vessel	Human	In vascular endothelial cells.	Okada et al., 2021
TMPRSS4	NA	Tongue (taste bud)	NA	NA	
		Tongue (mucosa)	Human	Suprabasal >basal	Huang et al., 2021
		Salivary glands	Human	TMPRSS4 was expressed in salivary glands.	Huang et al., 2021
		Lips	NA	NA	
		Gingiva	Human	TMPRSS4 was expressed in gingival mucosa. details?	Huang et al., 2021
		Buccal mucosa	Human	Suprabasal >basal	Huang et al., 2021
		Soft palate	Human	Suprabasal >basal	Huang et al., 2021
		Tonsil	Human	Suprabasal >basal	Huang et al., 2021
		Blood vessel	NA	NA	
TMPRSS11D	NA	Tongue (taste bud)	NA	NA	
		Tongue (mucosa)	Human	Suprabasal >basal	Huang et al., 2021
		Salivary glands	Human	Enriched in mucosal keratinocytes.	Huang et al., 2021; Drozdzik and Drozdzik, 2022
		Lips	NA	NA	
		Gingiva	Human	Enriched in mucosal keratinocytes.	Huang et al., 2021
		Buccal mucosa	Human	Suprabasal >basal	Huang et al., 2021
		Soft palate	Human	Suprabasal >basal	Huang et al., 2021
		Tonsil	Human	Suprabasal >basal	Huang et al., 2021
		Blood vessel	NA	NA	
CTSB	Cleave S2’ site of S protein	Tongue (taste bud)	NA	NA	
		Tongue (mucosa)	NA	NA	
		Salivary glands	Human	Abundantly expressed in minor salivary glands. Broadly expressed in the epithelia.	Huang et al., 2021; Drozdzik and Drozdzik, 2022
		Lips	NA	NA	
		Gingiva	Human	Broadly expressed in the epithelia.	Huang et al., 2021
		Buccal mucosa	NA	NA	
		Soft palate	NA	NA	
		Tonsil	NA	NA	
		Blood vessel	NA	NA	
CTSL	Cleave S2’ site of S protein	Tongue (taste bud)	NA	NA	
		Tongue (mucosa)	NA	NA	
		Salivary glands	Human	Abundantly expressed in minor salivary glands. Broadly expressed in the epithelia.	Huang et al., 2021; Drozdzik and Drozdzik, 2022
		Lips	NA	NA	
		Gingiva	Human	Broadly expressed in the epithelia.	Huang et al., 2021; Okada et al., 2021
		Buccal mucosa	NA	NA	
		Soft palate	NA	NA	
		Tonsil	NA	NA	
		Blood vessel	NA	NA	
TMPRSS3	NA	Salivary glands	Human	Correlate with the expression of ACE2 in salivary glands.	Song et al., 2020
TMPRSS5	NA	Salivary glands	Human	Correlate with the expression of ACE2 in salivary glands.	Song et al., 2020
TMPRSS7	NA	Salivary glands	Human	Correlate with the expression of ACE2 in salivary glands.	Song et al., 2020

**FIGURE 2:**
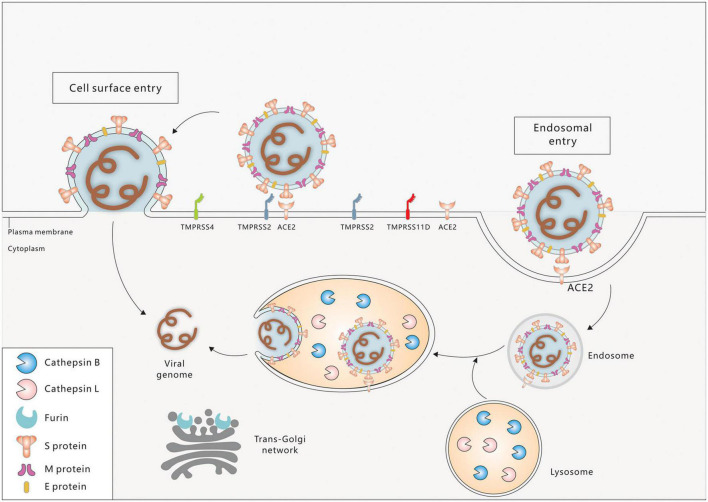
Cell entry mechanisms of SARC-CoV-2. Spike protein (S), envelope protein (E), and membrane protein (M) are three major proteins on the membrane of SARS-CoV-2. Cell surface entry and endosome entry are two distinct SARS-CoV-2 entry mechanisms. ACE2 is the primary receptor for SARS-CoV-2 entrance into the oral cavity. Virus binding to ACE2 triggers the conformational changes of the S1 subunit, exposing the S2’ site, which is then cleaved by the membrane protease TMPRSS2/4/11D (Cell surface entry pathway). In the absence of TMPRSS2, ACE2-virus complex is internalized *via* endocytosis into endosome/endolysosome where the S2’ site is cleaved by Cathepsin B/L (Endosome entry pathway). The fusion peptide is exposed and fuses with the cell membrane. The viral genome is then released into the cytoplasm of the host cell. In the virus producing cell, Furin cleaves the S1–S2 boundary in the *trans*-golgi network, contributing to the virus assembly and maturation.

Severe acute respiratory syndrome coronavirus-2 entry factors in the oral cavity include ACE2, TMPRSS2, TMPRSS4, TMPRSS11D, Furin, Cathepsin B (CTSB), Cathepsin L (CTSL), and others. During viral assembly and maturation, the S1/S2 site (multibasic site) of the S protein is recognized and cleaved by Furin, and the S1 and S2 subunits are subsequently stabilized by non-covalent binding. In the process of virus infection of target cells, the S protein binds to the ACE2 receptor on the target cell membrane, inducing a conformational change in the S protein and exposing the S2’ site. If TMPRSS2 is present on the target cell membrane, the S2’ site is cleaved by TMPRSS2 and initiates a membrane fusion process in which the virus fuses directly with the target cell membrane, followed by the release of viral RNA into the cytoplasm. If there are insufficient TMPRSS2 on the target cell membrane or the virus-ACE2 complex does not encounter TMPRSS2, the virus enters into the cell *via* clathrin-mediated endocytosis and forms an endosome. The S2’ site is then cleaved by cathepsins (CTSL/CTSB) and initiates the membrane fusion process, followed by the release of viral RNA into the cytoplasm (Figure 2; Li et al., 2003; Cai et al., 2020; Hoffmann et al., 2020; Lu et al., 2020; Jackson et al., 2022).

### 4.2. The possible mechanisms of dysgeusia

Patients with severe acute respiratory syndrome (SARS) and the middle east respiratory syndrome (MERS), beta-coronavirus infections, rarely suffered from dysgeusia (Pellegrino et al., 2020). The causes of dysgeusia in COVID-19 patients have been the subject of numerous investigations and theories.

#### 4.2.1. Dysfunction of taste buds

Severe acute respiratory syndrome coronavirus-2 may infect taste bud cells directly, resulting in dysgeusia (Mahmoud et al., 2021). According to previous studies, taste bud cells co-expressed ACE2 and TMPRSS2, which provided SARS-CoV-2 with receptors and hydrolases for invasion (Sakaguchi et al., 2020; Park et al., 2022). Doyle et al. (2021) found that ACE2 was expressed in human type II taste cells and SARS-CoV-2 could be replicated in type II taste cells through *in situ* hybridization. Additionally, it was revealed that the patient’s fungiform papillae taste stem cell layer has been damaged for several weeks, which could explain why dysgeusia lasted for a longer time. However, Wang Z. et al. (2020) showed that mice tongue papillae without taste buds had higher levels of ACE2 expression. Thus, SARS-CoV-2 may also potentially infect the squamous epithelial cells of the tongue, resulting in localized inflammation and edema and impairing the normal function of taste buds (Finsterer and Stollberger, 2020).

Additionally, the taste buds of mice had certain renin-angiotensin system (renin, angiotensinogen, and angiotensin-converting enzyme 1), and these constituents can locally create angiotensin II (Ang II), which can influence taste responses and be broken down into Ang 1–7 by ACE2. Accordingly, some studies hypothesized that patients’ taste buds may have less local Ang II degradation, which would lead to Ang II buildup and compromise the function of taste buds (Mariz et al., 2020).

#### 4.2.2. Dysfunction of the nervous system

Dysgeusia could be a complication of SARS-CoV-2 infection of the central nervous system (CNS) or peripheral nervous system (PNS). Dysgeusia occurring in COVID-19 patients was one of the most common neurological symptoms (Garg et al., 2020). ACE2 was expressed in some brain regions, such as motor cortex and posterior cingulate, nigra substance, ventricles, middle temporal gyrus, olfactory bulb, ventrolateral medulla, solitary tract nucleus, and vagus nerve, as well as some cells in CNS, including neurons, microglia, astrocytes, and oligodendrocytes, making the CNS a possible target organ for SARS-CoV-2 (Baig et al., 2020; Generoso et al., 2021). The autopsy examinations on COVID-19 patients revealed varied degrees of brain injury as well as the presence of viral RNA in the brain (Generoso et al., 2021; Maiese et al., 2021). Other studies have found that COVID-19 patients’ cerebrospinal fluid (CSF) contains SARS CoV-2 (Elmakaty et al., 2022). All the above evidences supported that SARS CoV-2 can target CNS. Two primary mechanisms for ACE2 related CNS infection have been found, namely hematogenous pathway and neural pathways. The former one is SARS-CoV-2 crossing the blood-brain barrier (BBB) *via* infecting the cerebral vascular endothelial cells or leukocytes. The latter refers to the virus traveling through the olfactory, trigeminal nerves (nasal cavity and nasopharynx) and the vagus nerve (lower respiratory tract) (Baig et al., 2020; Generoso et al., 2021). However, further studies are needed to determine whether SARS-CoV-2 infection of the CNS directly contributes to the development of dysgeusia in patients. Additionally, because SARS-CoV-2 is neurotropic, it may directly harm the cranial nerves (CN VII, CN IX, and CN X) responsible for transmitting taste (Lozada-Nur et al., 2020).

Impaired synaptic transmission may also contribute to dysgeusia. The neurotransmitters dopamine and 5-hydroxytryptamine (5-HT), both essential for synaptic transmission (including taste), are produced by the enzyme aromatic L-amino acid (DOPA) decarboxylase. However, SARS-CoV-2 may suppress the expression of dopamine decarboxylase in target cells, resulting in lower levels of dopamine and 5-HT that might impair regular synaptic transmission and cause dysgeusia (Finsterer and Stollberger, 2020).

#### 4.2.3. Olfactory dysfunction

Olfactory dysfunction (OD), one of the most common sensory dysfunction in patients with COVID-19, may also be one of the important causes for dysgeusia in patients (Mehraeen et al., 2021). About 41.5% of patients had both dysgeusia and OD as their primary symptom (Samimi Ardestani et al., 2021). The brain integrates the taste, smell, texture, temperature, appearance or sound of food or drink to form flavor. The insula, caudal orbitofrontal cortex (OFC), and anterior cingulate cortex (ACC) of the brain showed overlapping activation in response to independently presented tastes and scents, indicating that these areas may be crucial in the integration of taste and smell. Interestingly, putative primary gustatory areas occasionally respond to olfactory stimuli, whereas primary olfactory cortex does not seem to respond to gustatory stimuli. The integration of taste and smell in the insula, OFC, and ACC with other brain regions is also influenced by the olfactory delivery patterns and prior exposure to taste/smell combinations (Small et al., 2004; Landis et al., 2005; Small and Prescott, 2005; Hannum et al., 2018; Olofsson and Freiherr, 2019). Therefore, COVID-19 individuals who experience OD may also experience dysgeusia as a result of decreased olfactory delivery without stimulation of the gustatory cortex in the brain or defective integration of smell and taste.

### 4.3. The possible mechanisms of xerostomia

Salivary gland lesions could be caused by SARS-CoV-2 infection since ACE2 and TMPRSS2 were expressed in the ductal epithelium, serous acini, and mucous acini of the salivary glands. According to the research by Wang C. et al. (2020) SARS-CoV-2 can attack salivary glands *via* binding to the ACE2 receptor, leading to acute sialadenitis. Subsequently, the salivary glands may be repaired through fibroblast proliferation and fibrous connective tissue formation. However, this will also cause fibrosis of acinar cells and salivary gland ducts, resulting in a decrease in salivary secretion and obstruction of the salivary ducts (Wang C. et al., 2020). This theory offered a potential explanation for how salivary gland lesions manifest in COVID-19 patients. Chronic sialadenitis was the most prevalent histological alteration in infected salivary glands (Huang et al., 2021). They also discovered immune cells in the salivary glands, which suggested that sialadenitis was closely related to T cell responses. Bruno et al., also discovered morphological alterations in the epithelial cells and acinar cells of infected salivary gland (Matuck et al., 2021). The results stated above suggested that SARS-CoV-2 might infect salivary glands and trigger localized inflammation and immunological reactions. Additionally, increased mouth breathing and reduced salivary gland function were brought on by COVID-19 patients’ impaired nasal breathing, which in turn caused secondary symptoms such as xerostomia (Brandão et al., 2021).

### 4.4. The possible mechanisms of oral mucosal lesions

Since oral epithelial cells have high levels of ACE2, it is possible that SARS-CoV-2 might directly invading oral epithelial cells (Xu H. et al., 2020; Huang et al., 2021). Huang et al. (2021) discovered that SARS-CoV-2 may also infect the basal cells, suprabasal cells, and differentiated cells of the oral mucosal epithelium. Additionally, compared to other tissues, the oral mucosal epithelium has a low risk of contracting SARS-CoV-2 (Sapkota et al., 2022). However, it is unknown if oral mucosal lesions in patients are driven on by a direct infection with SARS-CoV-2 (Erbaş et al., 2022). The causes of oral mucosal lesions may also be related to oral cavity local immune responses, fungus infections, drug side effects, injuries caused by medical devices, vasculitis, microcirculation issues, etc (Viner and Whittaker, 2020; Amorim dos Santos et al., 2021b; La Rosa et al., 2021; Orilisi et al., 2021; Mohseni Afshar et al., 2022). Cell vacuolization, inflammatory cell infiltration, thrombosis, hemorrhage, necrosis, and other pathological abnormalities of the oral mucosa in COVID-19 individuals are additional oral mucosal abnormalities (Silveira et al., 2022). However, HPV infection was a potential source of these pathological manifestations (Hajdu, 2006).

## 5. Factors affecting SARS-CoV-2 infection

### 5.1. Periodontal pathogens

Periodontal infections are the common cause of the oral condition known as periodontitis. It has been revealed that 49.4% of COVID-19 patients had severe periodontitis (Anand et al., 2022). It was discovered that periodontal pathogens could impact affect the infectivity of SARS-CoV-2. Fusobacterium nucleatum, a periodontal infection, has been shown to boost ACE2 expression in A549 lung epithelial cells, according to Takahashi et al. (2021a). Despite the fact that this study did not show that Fusobacterium nucleatum could cause an increase in ACE2 expression in oral epithelial cells, it did provide compelling proof that periodontal infections might accelerate SARS-CoV-2 infection. In addition, Sena et al. (2021) reported that Porphyromonas gingivalis lipopolysaccharide (PgLPS) or inflammatory factors/mediators [e.g., interleukin-1β (IL-1β), tumor necrosis factor-α (TNF-α), and PGE2], derived from Porphyromonas gingivalis, could alter the expression levels of ACE2 and TMPRSS2 in human gingival fibroblasts. Some investigations showed that the S protein could be cleaved by the proteases produced by periodontal infections, increasing the infectivity of SARS-CoV-2 (Imai and Tanaka, 2021; Takahashi et al., 2021b).

Marouf et al. (2021) found that patients with periodontal illnesses are more vulnerable to COVID-19-related problems. SARS-CoV-2 was found in gingival crevicular fluid in roughly 63.64% of COVID-19 patients (Gupta et al., 2021). SARS-CoV-2 may spread through the periodontal tissues’ capillaries, promoting systemic infection (Badran et al., 2020; Bao et al., 2020; Drozdzik and Drozdzik, 2022). Furthermore, periodontal bacteria may be found in the bronchoalveolar lavage fluid of COVID-19 patients (Shen et al., 2020), and some research suggested that periodontal pathogens could worsen the symptoms of pneumonia or result in increased levels of systemic inflammatory cytokines (Nagaoka et al., 2014; Benedyk et al., 2016; Takahashi et al., 2021a). However, the precise processes through which periodontal disease affects the severity of COVID-19 are yet unclear.

### 5.2. Saliva

With sensitivity and specificity of 94.4 and 97.6%, respectively, saliva can be used as one of the dependable samples to diagnose COVID-19 in patients who are still in the early stages of the illness (Vaz et al., 2020). Chen L. et al. (2020) showed that saliva samples from severe patients contained more live viruses. However, a number of studies showed that saliva may offer some protection against the SARS-CoV-2. Immunoglobulin A (IgA), immunoglobulin M (IgM), and immunoglobulin G (IgG) antibodies against the S protein were present in the saliva of the individuals (Isho et al., 2020). IgM and IgG levels in saliva might be used to measure the immune response to SARS-CoV-2. Secretory IgA (SIgA), which can not only cross-react with the S1 subunit of the S protein but also stop the S protein from binding with the ACE2 receptor, was discovered by Tsukinoki et al. (2021) in the saliva of certain uninfected individuals. Lactoferrin, lysozyme, peroxidase, etc., in saliva could operate as general immunological defenses against SARS-CoV-2 infection (Tsukinoki et al., 2021). However, Exfoliated epithelial cells from COVID-19 patients’ saliva were found to be capable of sustaining SARS-CoV-2 infection and replication in a histology research (Huang et al., 2021). However, these exfoliated epithelial cells might have a local protective role against SARS-CoV-2 infection in the mouth (Drozdzik and Drozdzik, 2022).

### 5.3. Abnormal oral tissues

Sapkota et al. (2022) discovered that the expression level of ACE2 in oral squamous cell carcinoma cells and oral dysplasia tissues did not differ significantly from normal oral tissues whereas Furin expression rose and TMPRSS2 expression considerably decreased. However, it is still unclear how these changes may affect SARS-CoV-2 entry into host cells (Sapkota et al., 2022).

### 5.4. Oral health management during COVID-19 pandemic

It has been demonstrated that the SARS-CoV-2 spreads through spit droplets produced by talking, sneezing, and breathing (To et al., 2020; Xu R. et al., 2020). According to the research by Herrera et al. (2020) COVID-19 severity and viral excretion may be related to the amount of SARS-CoV-2 in the oral cavity. It has been demonstrated that gargling and tooth cleaning can lessen oral virus load (Mateos-Moreno et al., 2021). Therefore, by lowering the viral load in the mouth, oral healthcare may limit viral transmission. Patients should routinely wash their teeth, gargle, and use surgical masks or N95 masks in order to reduce the spread of SARS-CoV-2. Medical personnel should actively treat patients with periodontal diseases, sanitize the air in the facility to prevent saliva droplets from spreading, and pay attention to reducing the production of aerosols during oral surgeries in addition to wearing masks.

## 6. Conclusion

The oral signs, which mostly present as dysgeusia, xerostomia, and oral mucosal lesions that may be directly derived from SARS-CoV-2 or secondary COVID-19 lesions, may be helpful for the early diagnosis of individuals with COVID-19. The precise mechanics, meanwhile, are still not completely understood. Additionally, the infectivity of SARS-CoV-2 may be significantly influenced by factors such periodontal diseases, saliva, and abnormal oral tissues, necessitating the monitoring of patients’ oral health. Further investigation should be made into the diagnosis of COVID-19 using in-depth analysis of patients’ oral symptoms and associated processes.

## Author contributions

WL and FG drafted the manuscript. WL, XW, and OS revised the manuscript and prepared the table and figures. NQ and XC contributed to the literature review. KT and CZ participated to the study design. MZ and OS initiated the study and revised the manuscript. All the authors read and approved the final version of the manuscript.
